# Vision, Cognitive Impairment, and Dementia in the Aging, Demographics, and Memory Study

**DOI:** 10.1093/geroni/igab046.594

**Published:** 2021-12-17

**Authors:** Joshua Ehrlich, Bonnielin Swenor, Yunshu Zhou, Kenneth Langa

**Affiliations:** 1 University of Michigan, University of Michigan, Michigan, United States; 2 Johns Hopkins School of Medicine, Baltimore, Maryland, United States; 3 University of Michigan, Ann Arbor, Michigan, United States

## Abstract

Vision impairment (VI) is common in late-life and may be a modifiable risk factor for cognitive decline and dementia. In this study, using data from the population-based Aging, Demographics and Memory Study (ADAMS), we analyzed the association of VI with cognitive impairment no dementia (CIND) and dementia. We found that VI (binocular presenting acuity <20/40) was significantly associated with incident CIND (OR=3.5, 95% CI=1.4-8.9, p=0.008) and dementia (OR=1.8, 95% CI=1.0-3.1, p=0.040) after adjusting for age. However, among those with CIND, VI was not associated with dementia (OR=0.9, 95% CI=0.4-1.8, p=0.733). The association between VI and CIND remained significant in models fully adjusted for demographic and health factors (OR=2.7, 95% CI=1.0-7.5, p=0.049). We conclude that VI is associated with development of CIND but not with subsequent onset of dementia. These findings suggest that the association between VI and dementia is driven by the elevated risk of dementia among those with CIND.

